# Pediatric Oral Health Service Access in Racial/Ethnic Minority Neighborhoods: A Geospatial Analysis in Washington, D.C., USA

**DOI:** 10.26502/droh.0073

**Published:** 2024-03-06

**Authors:** Minxuan Lan, Eric Niu, Meirong Liu, Sadiyah Anderson, LaToya Barham, Tanya Greenfield, Gail Cherry-Peppers, Xinbin Gu

**Affiliations:** 1 Department of Geography and Planning, The University of Toledo, Toledo, OH, USA 43606; 2 Livingston High School, Livingston, NJ, USA; 3 School of Social Work, Howard University, Washington, DC, USA; 4 School of Art of Science, Howard University; 5 College of Dentistry, Howard University, Washington, DC, USA

**Keywords:** Oral health, Pediatric dentistry, Oral health disparity, Dental care, Geospatial analysis

## Abstract

Oral health plays a crucial role in overall well-being. One of the goals set by the US Department of Health and Human Service, Healthy People 2030 is to reducing dental caries in children and adolescents. The study aims to investigate the accessibility of pediatric dental care in neighborhoods with mixed-race and predominantly African American populations in the Washington District of Columbia (DC) area. Our objective is to uncover and highlight the disparities that exist in pediatric dental care within these communities. We have specifically examined the geographic and socio-demographic aspects of pediatric dental care facilities, utilizing geospatial tools such as modeling and mapping, as well as data from the clinical database at Howard University College of Dentistry. The detailed analysis of dental care access revealed significant disparities among various Wards in the region. Specifically, Wards 5, 7, and 8 stood out as having both the highest concentrations of African American residents and the lowest availability of pediatric dentistry providers when compared to the more affluent Wards 1, 2, and 3. Howard University College of Dentistry’s pediatric dentistry department played a crucial role in providing dental care services to the community. Over the course of the year 2022, they recorded a total of 3,855 visits from residents of the DC area. Notably, a substantial portion of these visits, specifically 1,566 visits, were from individuals residing in Wards 5, 7 and 8. This data underscores the significant demand for pediatric dental services in these underserved communities and highlights the importance of addressing the disparities in access to care.

## Introduction

Pediatric oral health is a crucial factor that must be focused on when tackling oral health disparities. The US Surgeon General’s report from 2021, “Oral Health in America: Advances and Challenges,” highlighted the significance of oral health for children, acknowledging it as a crucial aspect of a child’s overall health and the need for defecated effort to improve oral health outcomes from a young age [1]. The importance of initiating proper oral health practices during infancy and childhood is emphasized as foundational for sustaining healthy habits and achieving positive oral health outcomes into adulthood. The report draws attention to the marked disparities in oral health among children, particularly affecting those from economically disadvantaged and minority backgrounds, with these disparities linked to a variety of factors including access to healthcare, socioeconomic status, and educational opportunities. Although there have been strides toward improving access to dental care for children through broader dental insurance coverage and the incorporation of dental services into pediatric healthcare, significant barriers still hinder comprehensive access to routine and preventive dental care for all children.

While i’s often assumed that children, being young and generally healthy, may not require as much attention to oral care, they are at risk for the same dental issues that affect adults. Moreover, disparities in oral health are a significant concern for children. Dental caries, as one of the leading chronic diseases, impacts around 70% of children in disadvantaged families across the globe. This condition is more prevalent among ethnic minorities, children living in rural settings, and those from lower socio-economic backgrounds [2].

Research into pediatric dental diseases and disparities is not as extensive as that for adult dental diseases, indicating a need for more focused studies on children’s oral health disparities. There is a critical need for additional research in this area to enhance awareness and prompt changes in health policy, allocation of resources, education, workforce development, and community outreach efforts. Such efforts aim to improve both the quality and the accessibility of pediatric dental services. This study is designed to assess the availability of dental care for children in mixed-race and predominantly African American communities in the Washington, D.C. area. This study is a first step in highlighting the pediatric oral health disparities that exist, specifically in the Washington, D.C. area. We want to take a look at a plethora of factors contributing to possible pediatric oral health disparities using geospatial tools. The city is split into eight smaller legal subsections known as Wards, each having distinct sociodemographic components. The Anacostia River separates the city not just physically, but also economically and ethnically, with wealth concentrated west of the river (Wards 1-6) and poverty concentrated east of the river (Wards 7 and 8) [3]. The poverty rate for children east of the Anacostia River is 46%, compared to 13% for children in the remainder of the city. Additionally, 71% of disadvantaged children in DC live east of the Anacostia River [4].

Bronfenbrenner’s Ecological Systems Framework places humans in the context of their environment, where the interactions of different systems can impact individual health, well-being, and behaviors [5]. According to this theory, access to care is determined by larger contextual variables, such as the environment. We believe that the closer the services are geographically located, the more likely an individual is to use them. Based on this notion, we want to investigate youth residents’ access to pediatric oral health providers in the Washington District Colobus (D.C.), posing the study question: Is the geographical accessibility of pediatric oral health services less for racial/ethnic minority communities? We begin by looking at the spatial distribution of pediatric oral health services in the city. Then, we analyze how neighborhood variables, such as racial composition and poverty rates, may be associated with people’s access to oral health services.

Overall, this study aims to shed light on the disparities in pediatric dental care access across diverse communities within the Washington, D.C. area and assess the impact of the dental clinic at Howard University College of Dentistry in addressing these disparities.

## Materials & Methods

### General Data collection

The data was collected on pediatric dentists in Washington D.C. using google.maps.com on June 21, 2023. Information regarding poverty, gender, and ethnicity was obtained from the 2020 US Census Bureau, which is the latest available census data. Data from the clinical activities conducted by the Howard University College of Dentistry (HUCD)’s Department of Pediatric Dentistry in 2022 were gathered to assess the impact of HUCD’s pediatric dentistry program. The data was archived in the Axium system. By utilizing patients’ home zip codes, we aligned the data with the respective D.C. Wards.

### Geospatial Analyses

Data compilation and spatial analysis were conducted using ArcGIS Desktop (ArcMap) 10.8.1. This software presents geographic data through a layered map view. Developed by Esri (Environmental Systems Research Institute Inc.), a global frontrunner in GIS software, location intelligence, and mapping, ArcGIS Desktop is a predominant tool in the GIS domain, embraced by academic bodies, governmental organizations, and the private sector.

### Geocoding Process

Utilizing Geocoding, we transform textual addresses, such as “2440 M Street Northwest, Washington, DC 20037”, into their corresponding geographic coordinates, like “38.90517737, −77.05220463”. This allows for precise mapping on ArcMap. All 58 pediatric dentists’ addresses were successfully geocoded, facilitating an accurate count of pediatric dentists across the Wards in Washington D.C.

### Kernel Density Estimation (KDE)

Through KDE, we can study the distribution of these 58 pediatric dentists across a spatial domain. KDE calculates a magnitude-per-unit area from dots using a kernel function to fit a smoothly tapered surface to each point [6,7]. The result of KDE can reveal clustering patterns and identify isolated data points [8]. For instance, it can answer questions like: Are many pediatric dentists located near to one another? Or are they spaced out?

### Cluster and Outlier Analysis

This analysis pinpoints significant hot spots, cold spots, and spatial outliers based on the Anselin Local Moran’s I statistic. This method determines the significance with a p-value less than 0.05 [8].

## Results and Discussion

### Distribution of Pediatric Dental Clinics in Washington, D.C. Area

We collected the addresses of 58 pediatric dentists in Washington D.C. from google.maps.com on June 21, 2023. The socioeconomic data, including details about poverty, gender, and ethnicity, were obtained from the 2020 US Census Bureau, which represents the latest census data. Washington, D.C. stands as the national’s capital and is unique as the only federal district of the United States. The city is located on the east bank of the Potomac River, surrounded by Virginia and Maryland. As of 2020, the city housed a population of 701,974, comprising 41% white, 45% African American, 4% Asian, and 10% from other ethnicities. Hispanic or Latino individuals made up 11% of the population. Males constituted about 47% of the total. Notably, approximately15% of the inhabitants fall below the US Census Bureau’s defined poverty threshold. To delve into the spatial distribution of pediatric dental clinics and to understand their relation to the diverse socioeconomic factors at both Ward and census tract scales, we employed geospatial analysis methods.

**Figure 1:**
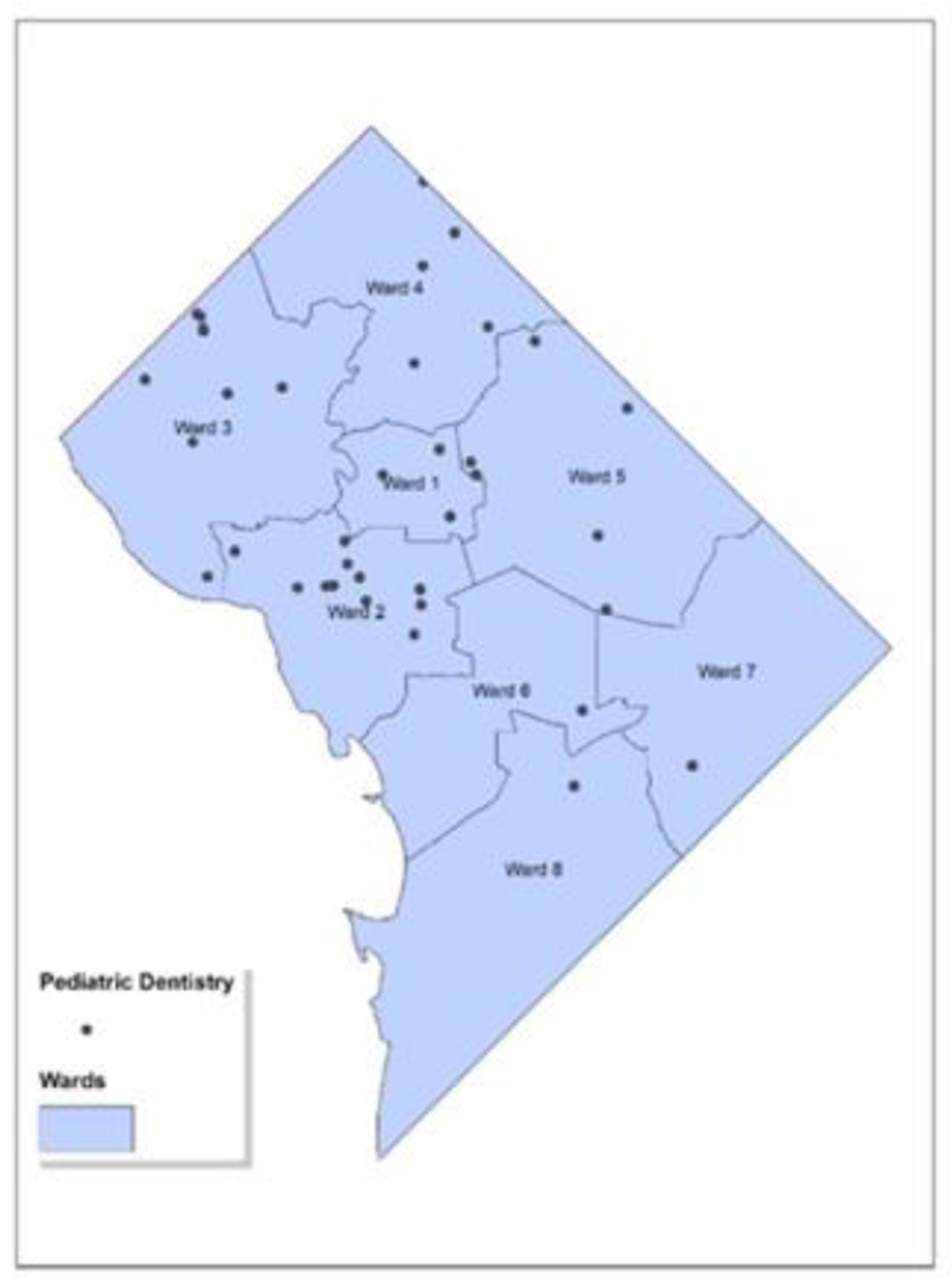
Geocoded Map of Pediatric Dental Clinics in Washington, D.C. The map illustrates the geographic distribution of pediatric dental clinics across Washington, D.C. The locations of the clinics are denoted by black dots. The map is segmented into areas labeled as Wards 1 through 8, which are typical divisions used for city planning and the allocation of services.

Figure 1 presents a map highlighting the 58 pediatric dentists in Washington, D.C., overlaid with labeled Ward boundaries. The black dots on the map indicate the locations of these pediatric dentists. It is important to note that since multiple dentists can operate from the same address (with different suite numbers), some dots may overlap. Such a map can be instrumental in assessing the availability of pediatric dental care throughout the various regions of the city.

An initial observation indicates that Wards 6-8 have a sparser presence of these service providers. To verify this observation, we carried out further spatial analyses to better comprehend the dispersion and spread of pediatric dentists.

**Figure 2:**
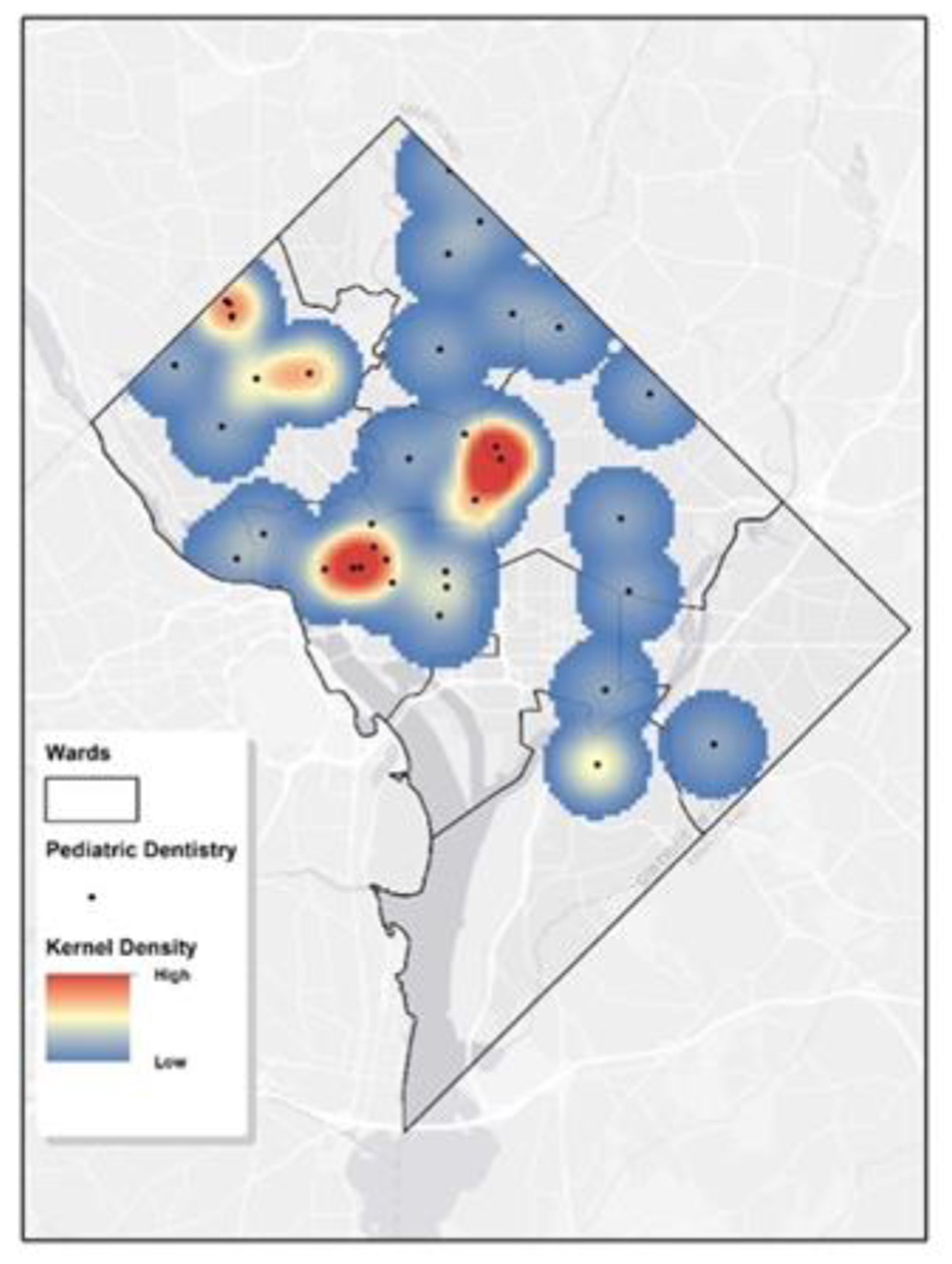
Kernel Density Estimation of Pediatric Dental Services in Washington, D.C.The map displays a geographic heatmap that employs kernel density estimation to convey the concentration of pediatric dental services throughout Washington, D.C. On this map, areas marked in red and yellow denote regions with a higher density of pediatric dental clinics, whereas blue areas represent regions with a lower density of such services.

We employed KDE to examine the spatial distribution pattern of the 58 pediatric dentists. This analysis can highlight areas of clustering or sparse distribution. Figure 2 displays the KDE visualization for these pediatric dentists in D.C. The cell size is 400 feet, and the search radius is 4000 feet. These parameters were chosen based on the study unit dimensions and average Ward size. A warmer hue indicates denser clustering, with red signifying high concentrations of pediatric dentists and blue denoting sparser areas. This visual tool is beneficial for pinpointing regions with robust dental service availability for children and recognizing those that may require more resources. Wards 1-3 and Ward 5 near the west edge, show the densest presence of pediatric dentists. On the other hand, Ward 6-8 and majority of Ward 5 exhibit notably lower densities. These findings are consistent with the observation in figure 1. The College of Dentistry clinic at Howard University is situated in Ward 1, near the western edge of Ward 5. Consequently, this area exhibits a high density.

**Figure 3:**
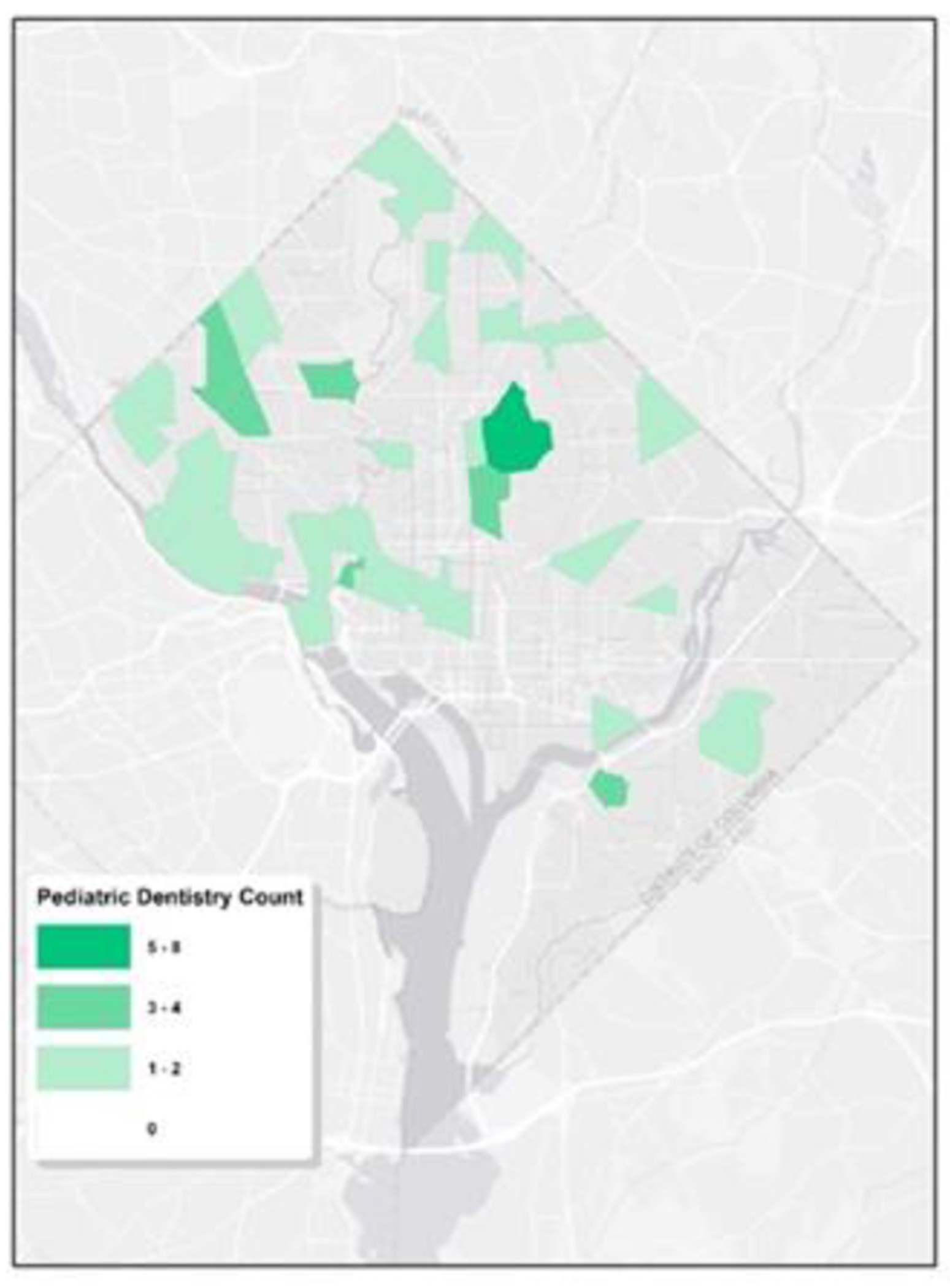
Pediatric Dentistry Count by Census Tract. The map quantifies pediatric dental clinics across Washington, D.C., based on census tracts. Different intensities of green are used to signify the number of clinics, with deeper greens indicating a larger count.

To delve deeper into the spatial trends of pediatric dentistry in Washington, D.C., we utilized census tract data to enumerate the presence of pediatric dentists. Figure 3 shows a visual representation, highlighting the distribution of pediatric dentists across the different census tracts, as well as limited dental resources for children. It’s evident that Wards 1, 2, and 3 are densely populated with pediatric dentists, presenting a stark contract to Ward 6, 7, and 8, where a significant number of tracts are devoid of any pediatric dental professionals. Clearly, the distribution of them is highly uneven. The uneven dispersion reveals that while some tracts are fortunate to have as many as eight pediatric dentists, there are multiple other tracts where the community doesn’t have access to even a single one.

**Figure 4:**
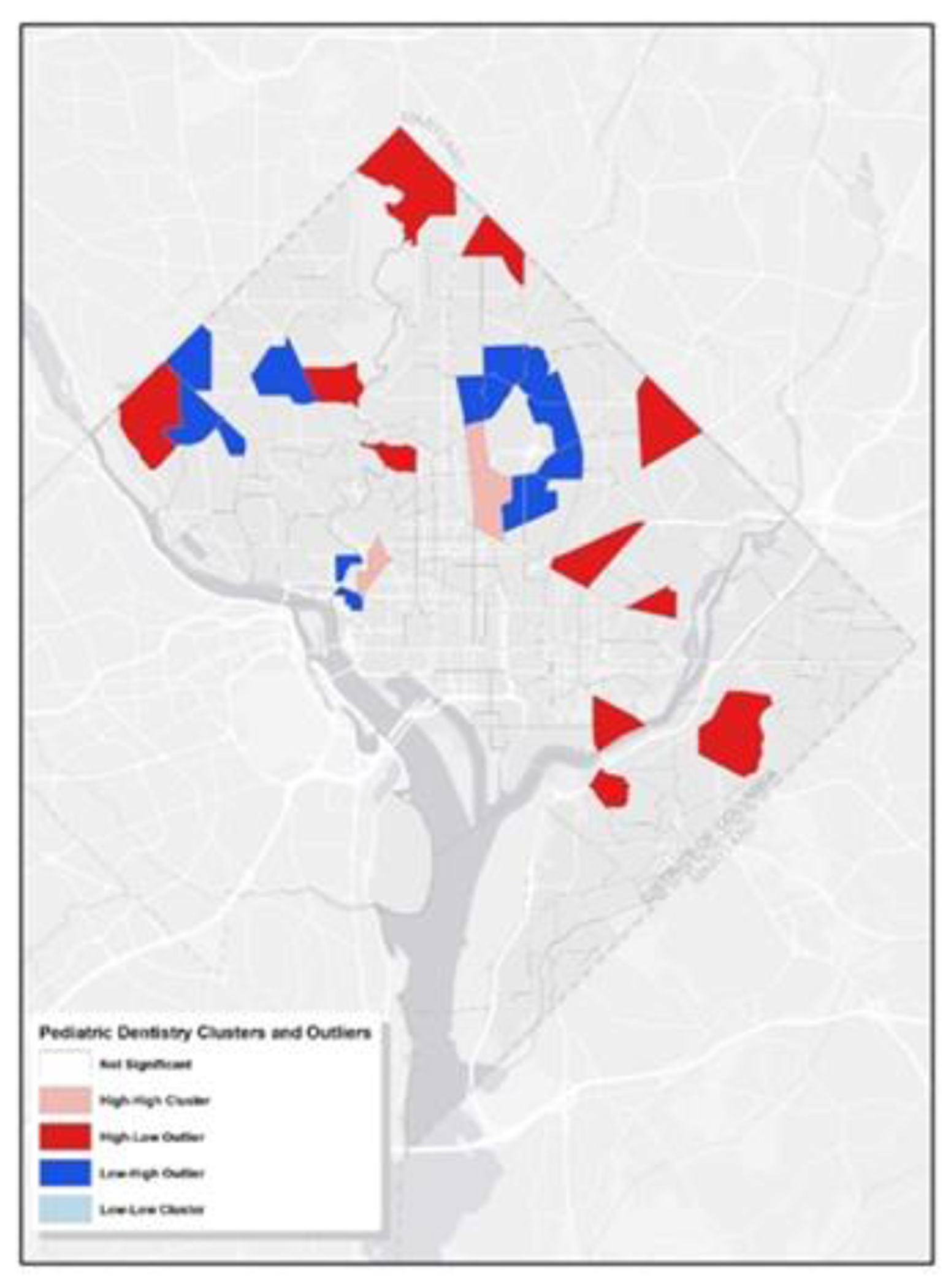
Clusters and Outliers of Pediatric Dentists. The map illustrates the geographic distribution of pediatric dental offices in Washington, D.C. It employs arrows in two colors to denote significant concentrations of dental clinics (presumably indicated by blue) and solitary, standalone clinics (presumably indicated by red).

Additionally, we employed Cluster and Outlier Analysis to pinpoint statistically significant hot spots, cold spots, and spatial outliers with a p-value greater than 0.05. This visualization is instrumental in pinpointing areas densely populated with dental services for children and areas where such services are notably sparse, providing insights into the patterns of pediatric dental care accessibility across the city. As illustrated in figure 4, the pink regions represent tracts with a high concentration of pediatric dentists, surrounded by similar high-concentration tracts (cluster). The red tracts indicate areas with many pediatric dentists but neighbored by areas with fewer pediatric dentists (outlier). Conversely, the dark blue tracts have a lower number of pediatric dentists yet are surrounded by tracts with a higher count of pediatric dentists (outlier). Comparing this with figure 1, it is evident that the clusters predominantly exist in Wards 1 and 2. On the other hand, the city’s peripheries, notably Ward 5, 7, and 8, encompass most outliers. This further suggests the uneven distribution of pediatric dentistry across the examined region.

### Relationship of Pediatric Dentistry and Socioeconomic Factors

**Figure 5:**
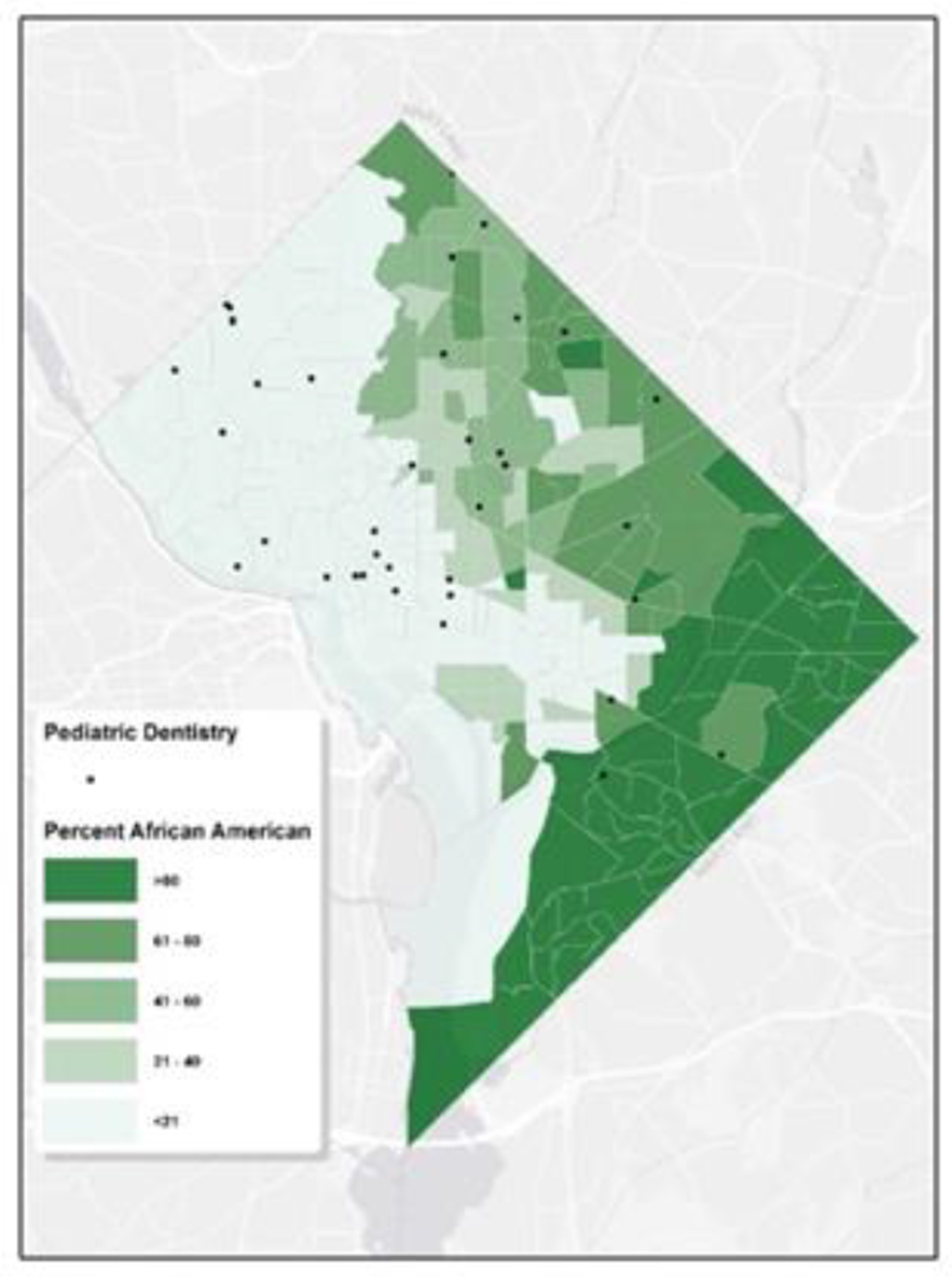
Map of Pediatric Dental Services & African American Tract-Level Characteristics. The map depicts the distribution of pediatric dental clinics in Washington, D.C. in relation to African American population characteristics at the census tract level. The map is shaded in varying degrees of green, where darker shades indicate a larger African American population within those tracts. The locations of the pediatric dental clinics are denoted by black dots.

Washington D.C., like many urban areas in the U.S., has experienced various socio-economic, political, and historical factors that have determined its demographic layout. Historically, during significant migration phases, African Americans predominantly settled in neighborhoods now recognized as Ward 7 and 8. Figure 5 clearly exhibits the disparities in access to pediatric oral healthcare for African Americans on a census tract basis. This kind of map can be valuable for public health officials and policymakers to understand the alignment between dental service locations and demographic concentrations. While Ward 5, 7, and 8 are home to the densest African Americans populations, they are starkly underserved in terms of pediatric dentistry services, especially towards the southern boundary encompassing Wards 7 and 8.

**Figure 6:**
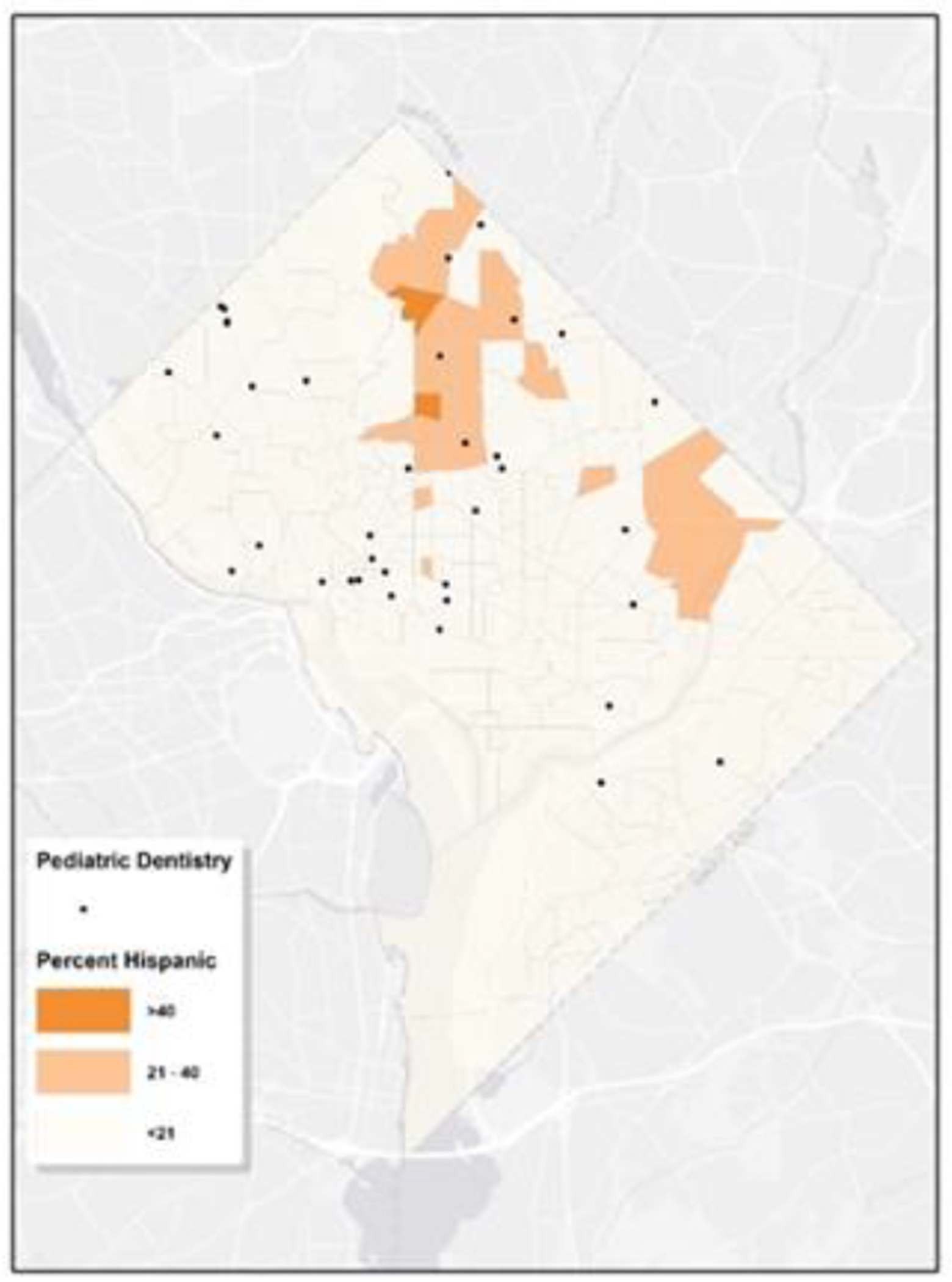
Map of Pediatric Dental Services & Hispanic Tract-Level Characteristics. The map illustrates the locations of pediatric dental services in relation to the Hispanic population across various census tracts in Washington, D.C. The map employs a palette of orange hues to signify the density of the Hispanic population within the tracts. The locations of the pediatric dental clinics are denoted by black dots.

**Figure 7:**
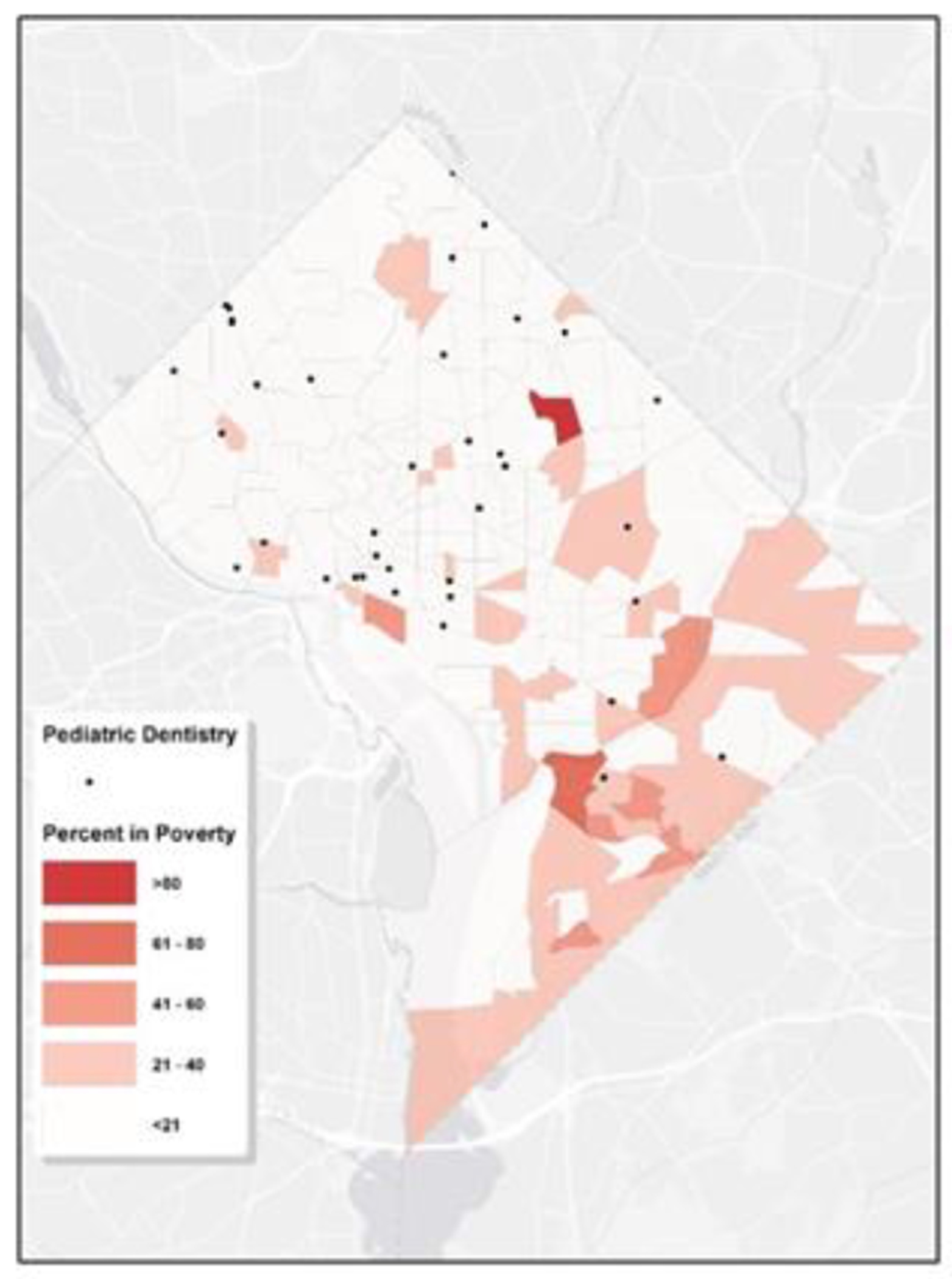
Map of Pediatric Dental Services & Poverty Tract-Level Characteristics. The geographic distribution map illustrates the spread of pediatric dental facilities in relation to areas with varying economic profiles within a city, using varying intensities of red to represent the data. Darker hues signify higher poverty rates in those census tracts. The locations of the pediatric dental clinics are denoted by black dots.

Figure 6 illustrates that communities with a predominant Hispanic/Latino population, are mainly situated in the northern regions, specifically in Wards 1, 4, and 5. These areas appear to have relatively better access to pediatric dental care. Figure 7 shows the proportion of residents living in poverty within each tract. While signs of poverty can be observed citywide, Wards 5, 7, and 8 stand out with higher concentrations. In tracts where poverty rates are elevated, access to pediatric dental care appears to be notably limited. Within Washington D.C., the scarcity of pediatric dentistry in certain regions can be linked to socioeconomic disparities.

Historically, some Wards in D.C. have grappled with economic hurdles, which might affect residents’ capacity to afford dental care. This, in turn, might diminish the appeal for clinics to operate in these areas. Additionally, a limited number of specialists might be another contributing factor, with fewer pediatric dentists inclined to establish their practice in these less affluent areas, expecting diminished returns.

### Impact of Pediatric Dental Care Services at Howard University College of Dentistry

**Figure 8:**
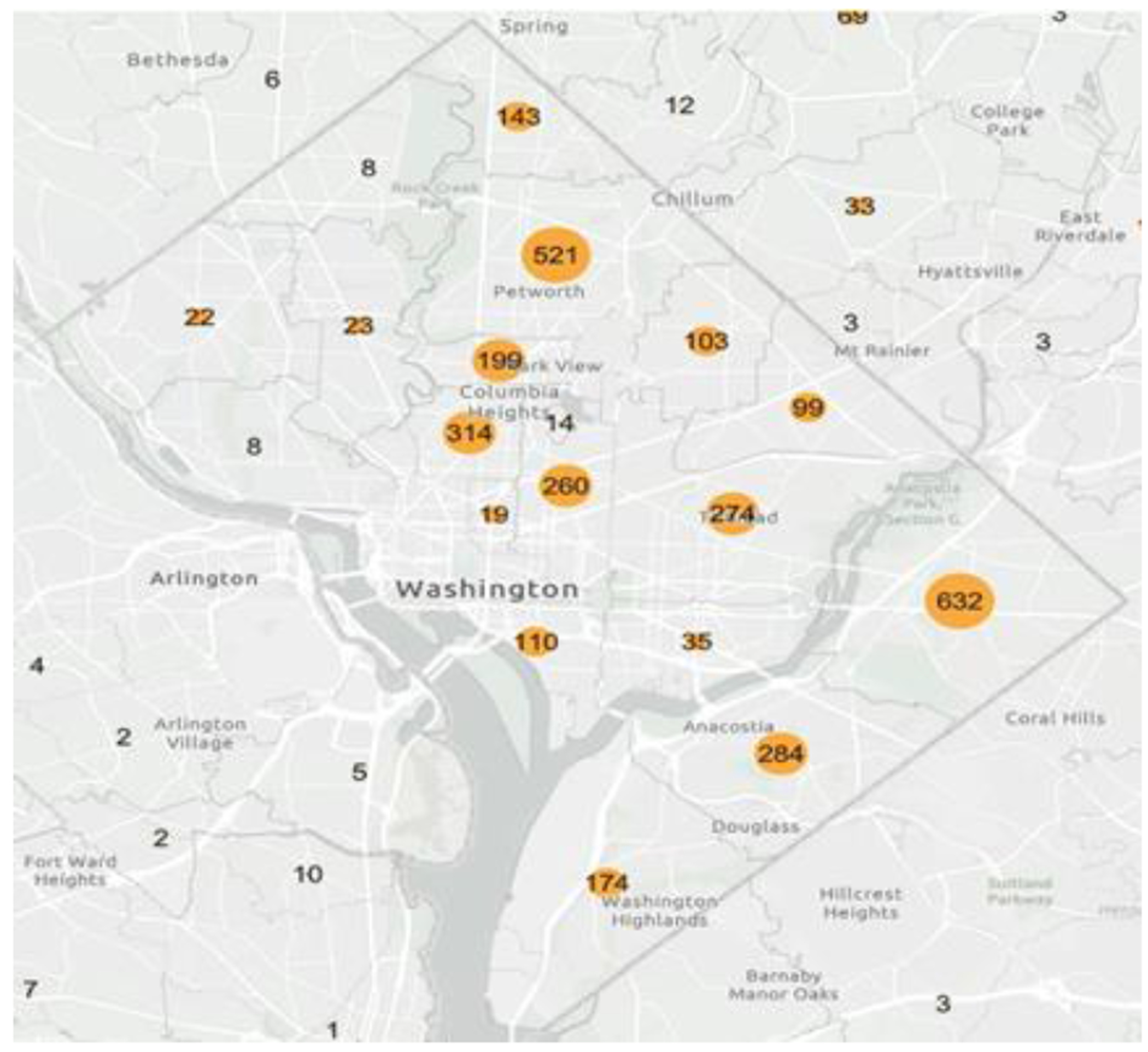
Map of Pediatric Dental Services provided by Howard University College of Dentistry, 2022. The map provides a visual representation of the patient outreach by Howard University College of Dentistry throughout Washington, D.C. and its adjacent regions, reflecting the extent of pediatric dental care delivered by the institution in the year 2022.

Situated in the nation’s capital, Washington D.C., Howard University has long been recognized as a premier historically black college and university (HBCU). Its College of Dentistry, in particular, stands as a beacon of hope for many medically underserved populations, offering essential oral health care services amidst a landscape of varying access to medical resources. Figure 8 presents an in-depth analysis of the pediatric dental services provided by Howard University College of Dentistry in 2022. Over that year, the dental clinic at the college registered a commendable 3,855 pediatric appointments from residents across D.C. A closer examination of these numbers shows distinct trends: of the total visits, 1,090 were from Wards 7 and 8 combined, 664 from Ward 4, and 476 from Ward 5. I’s noteworthy to mention that a significant majority of these patients were African Americans or Hispanics.

These wards, historically underserved and facing a myriad of socioeconomic challenges, have been left in a vulnerable position due to a palpable scarcity of pediatric dental facilities. Yet, the sheer volume of visits from these very wards to Howard’s dental clinic underscores the latent, unmet demand for such critical services. Furthermore, it serves as a testament to the university’s unwavering commitment to bridging healthcare disparities, particularly in the realm of pediatric dentistry.

## Discussion

Oral health is a crucial component of overall wellness, yet it frequently remains underemphasized and underserved [9]. The human body works like a machine, each individual part of human health is essential to the function of the whole. Oral health should not be overlooked, as regular oral checkups and cleanings play a key role in preventing and identifying problems early when they are simpler to manage. Neglected oral health can lead to serious systemic issues and even death [10]. Diseases like periodontal disease have been linked to a range of conditions including diabetes, metabolic syndrome, obesity, eating disorders, liver disease, cardiovascular disease, Alzheimer disease, rheumatoid arthritis, adverse pregnancy outcomes, and cancer [11]. Furthermore, poor dental health impacts productivity for both children and adults. Children suffering from dental pain or untreated infections are likely to perform poorly in school or miss school altogether, leading to approximately 50 million lost school hours annually. This not only affects the child’s education but also results in lost workdays for their caregivers [12-14].

In its fifth publication, the US Department of Health and Human Services through the Healthy People 2030 initiative, outlined 11 goals focused on improving oral health. These goals include decreasing dental cavities among children and teenagers, reducing the prevalence of untreated decay and gum disease in adults, and enhancing preventive measures such as water fluoridation, the use of dental sealants, conducting oral cancer screenings, and importantly, expanding access to dental care services [15]. While there has been notable progress in advancing dental technologies, treatments, and practices that benefit patients, especially those from underserved populations, significant health disparities remain [16,17]. Previous efforts to diminish these disparities, including those following the Affordable Care Ac’s implementation, have not shown substantial improvements, especially for individuals with disabilities and those in rural locales [18]. The issue of oral health inequity is widespread, affecting communities across the United States and globally, driven by various socioeconomic factors including income and racial disparities [19,20]. Furthermore, differences in ethnicity, gender, sexual orientation, and educational background also contribute to these disparities [16,21,22]. Environmental factors, such as the availability and accessibility of dental care services, further compound the issue. Clearly, the challenge of addressing oral health disparities is intricate, influenced by a broad array of social and environmental factors [23].

In conclusion, this research thoroughly investigates the inequalities in access to pediatric dental care across certain areas of the Washington, DC region. It highlights the urgent need for such services, especially in underserved communities, notably in Ward 7 and Ward 8. The Howard University College of Dentistry has made commendable efforts to address this issue by providing essential dental care to medically underserved populations, showcasing a proactive approach to a persistent problem. Nonetheless, broader systemic changes are required. Partnerships among government bodies, non-profits, and educational entities like Howard could lead to the development of more community-oriented dental programs. Furthermore, pushing for policies that ensure a fair distribution of dental resources and offering incentives for dental practices to operate in underserved regions may serve as effective strategies to diminish these access disparities.
